# The Host Protein CAD Regulates the Replication of FMDV through the Function of Pyrimidines’ *De Novo* Synthesis

**DOI:** 10.1128/jvi.00369-23

**Published:** 2023-05-10

**Authors:** Pu Yang, Yuncong Yuan, Yidan Sun, Bonan Lv, Hang Du, Zhou Zhou, Zhuang Yang, Xuemei Liu, Huimin Duan, Chao Shen

**Affiliations:** a College of Life Sciences, Wuhan University, Wuhan, China; b China Center for Type Culture Collection, Wuhan University, Wuhan, China; University of Kentucky College of Medicine

**Keywords:** FMDV, HDAC1, pyrimidines, AKT-mTOR signaling pathway, CAD

## Abstract

Foot-and-mouth disease virus (FMDV) is a single-stranded picornavirus that causes economically devastating disease in even-hooved animals. There has been little research on the function of host cells during FMDV infection. We aimed to shed light on key host factors associated with FMDV replication during acute infection. We found that HDAC1 overexpression in host cells induced upregulation of FMDV RNA and protein levels. Activation of the AKT-mammalian target of rapamycin (mTOR) signaling pathway using bpV(HOpic) or SC79 also promoted FMDV replication. Furthermore, short hairpin RNA (shRNA)-induced suppression of carbamoyl-phosphate synthetase 2, aspartate transcarbamylase, and dihydroorotase (CAD), a transcription factor downstream of the AKT-mTOR signaling pathway, resulted in downregulation of FMDV RNA and protein levels. Coimmunoprecipitation assays showed that the ACTase domain of CAD could interact with the FMDV 2C protein, suggesting that the ACTase domain of CAD may be critical in FMDV replication. CAD proteins participate in *de novo* pyrimidine synthesis. Inhibition of FMDV replication by deletion of the ACTase domain of CAD in host cells could be reversed by supplementation with uracil. These results revealed that the contribution of the CAD ACTase domain to FMDV replication is dependent on *de novo* pyrimidine synthesis. Our research shows that HDAC1 promotes FMDV replication by regulating *de novo* pyrimidine synthesis from CAD via the AKT-mTOR signaling pathway.

**IMPORTANCE** Foot-and-mouth disease virus is an animal virus of the *Picornaviridae* family that seriously harms the development of animal husbandry and foreign trade of related products, and there is still a lack of effective means to control its harm. Replication complexes would generate during FMDV replication to ensure efficient replication cycles. 2C is a common viral protein in the replication complex of *Picornaviridae* virus, which is thought to be an essential component of membrane rearrangement and viral replication complex formation. The host protein CAD is a key protein in the pyrimidines *de novo* synthesis. In our research, the interaction of CAD and FMDV 2C was demonstrated in FMDV-infected BHK-21 cells, and it colocalized with 2C in the replication complex. The inhibition of the expression of FMDV 3D protein through interference with CAD and supplementation with exogenous pyrimidines reversed this inhibition, suggesting that FMDV might recruit CAD through the 2C protein to ensure pyrimidine supply during replication. In addition, we also found that FMDV infection decreased the expression of the host protein HDAC1 and ultimately inhibited CAD activity through the AKT-mTOR signaling pathway. These results revealed a unique means of counteracting the virus in BHK-21 cells lacking the interferon (IFN) signaling pathway. In conclusion, our study provides some potential targets for the development of drugs against FMDV.

## INTRODUCTION

Foot-and-mouth disease is a viral disease of livestock that causes significant economic loss (1). The foot-and-mouth disease virus (FMDV) has a single-stranded positive RNA genome of approximately 8.5 kb encoding a large polyprotein that is processed into four structural proteins and eight nonstructural proteins (2). To replicate rapidly and efficiently in hosts, FMDV has evolved strategies to antagonize and evade the innate immune response. The FMDV structural proteins VP1 and VP3 and the nonstructural proteins L^pro^, 3A^pro^, and 3C^pro^ each regulate the innate immune response by different mechanisms (3–6). The 5′ untranslated region (UTR) of the FMDV genome consists of a highly structured S fragment of approximately 370 nucleotides and an internal polycytidine [poly(C)] of variable length. Downstream of the poly(C) (7), there is a pseudoknot region upstream of the internal ribosome entry site (IRES). IRES is a segment consisting of ~440 residues for internal initiation of protein synthesis in a CAP-independent manner (8). The 3′ UTR of the FMDV genome consists of 90 nucleotides with two stem-loop structures and a poly(A) region (9).

Eukaryotic cells replicate and transcribe their genome in the nucleus, whereas many RNA viruses and some DNA viruses carry out these functions in the cytoplasm. To efficiently replicate and protect themselves from host defenses, many viruses replicate and transcribe their genome within an organelle-like compartment in the cytoplasm (10). These compartments are often associated with subsequent stages of the viral replication cycle, including particle formation and viral budding (11). Recently, great progress has been made in characterizing the cytoplasmic replication compartments of positive-stranded RNA viruses such as FMDV. These viruses package their genomes as messenger-sense, single-stranded RNAs and replicate them through RNA intermediates (12). The viral replication complex regulates the exchange of substances inside and outside the organelle-like replication compartment during this process, allowing the components required for transcription, replication, and virus particle assembly to be maintained in high local concentrations inside the compartment.

Carbamoyl-phosphate synthetase 2, aspartate transcarbamylase, and dihydroorotase (CAD) is a multifunctional protein involved in the first three steps of pyrimidine synthesis. Structurally, CAD is a hexamer of 243-kDa polypeptide chains (13). The multiple functions of CAD result from the synergistic action of four domains, glutamine transferase (GATase), carbamoyl phosphatase II (CPSIIase), aspartate carbamoyl transferase (ATCase), and dihydroorotase (DHOase). The CPSIIase domain is made up of two highly homologous fragments, named CPSase A and CPSase B, which work together with the GATase domain to form a glutamine-dependent CPSase. Specifically, HCO^3−^ is transferred by the GATase domain to the CPSIIase domain to form carbamoyl phosphate (CP) with glutamine and ATP. The formation of CP is a rate-limiting step in nucleotide synthesis (14, 15). The ATCase domain has a catalytic homodimer that catalyzes the conversion of CP and aspartate to carbamoyl aspartate (CA-Asp) (16). The DHOase domain takes part in the reversible cyclization of CA-Asp to form dihydroorotic acid (DHO), the first cyclic compound in the *de novo* pyrimidine synthesis pathway (13, 17). DHO is reduced in the mitochondria by DHO dehydrogenase to orotate (18), which is finally transformed into the end product, UMP, by UMP synthase (19, 20).

Intracellular viruses and bacteria use the nucleotides synthesized by host cells for their growth and replication. As a result, pyrimidine biosynthetic enzymes have become a target for antiviral and antibacterial drug therapies. A study of primary human liver cells revealed that CAD is a key host factor in the life cycle of the hepatitis D virus (21). DHO dehydrogenase, which catalyzes the production of whey acids from DHO in the pyrimidine biosynthetic pathway, was an effective antiviral target for some small-molecule reagents (22). Activation of the UDP-glucose receptor enhanced neutrophil lung recruitment and stimulated interleukin 8 (IL-8) expression by human endometrial epithelial cells (23, 24). NOD2, a pattern recognition receptor of the nucleotide-binding oligomeric domain family, can sense conserved microbial-associated molecular patterns, trigger signaling cascades, and induce the secretion of proinflammatory cytokines, chemokines, and antimicrobial peptides. A recent study indicated that NOD2 could regulate its function by binding to the CPSase domain of CAD (25, 26). Inhibition of pyrimidine synthesis through the ATCase domain of CAD enhances the secretion of antimicrobial peptides by human cells. It is worth noting, however, that inhibition of pyrimidine synthesis has no direct antibacterial effect.

CAD is activated by ribosomal protein S6 kinase beta-1 (S6K1), a downstream effector of the AKT-mammalian target of rapamycin (mTOR) signaling pathway, which regulates a wide range of cellular processes, including survival, proliferation, growth, metabolism, angiogenesis, and cancer metastasis (27–29). Histone deacetylase 1 (HDAC1) is one of several upstream proteins that regulate the AKT-mTOR signaling pathway. We previously showed that HDAC1 was differentially expressed after acute FMDV infection (30, 31). Here, we describe a series of experiments to further investigate how HDAC1, the AKT-mTOR signaling pathway, and CAD form an axis that regulates FMDV infection.

## RESULTS

### HDAC1 contributes to FMDV replication.

**FIG 1:**
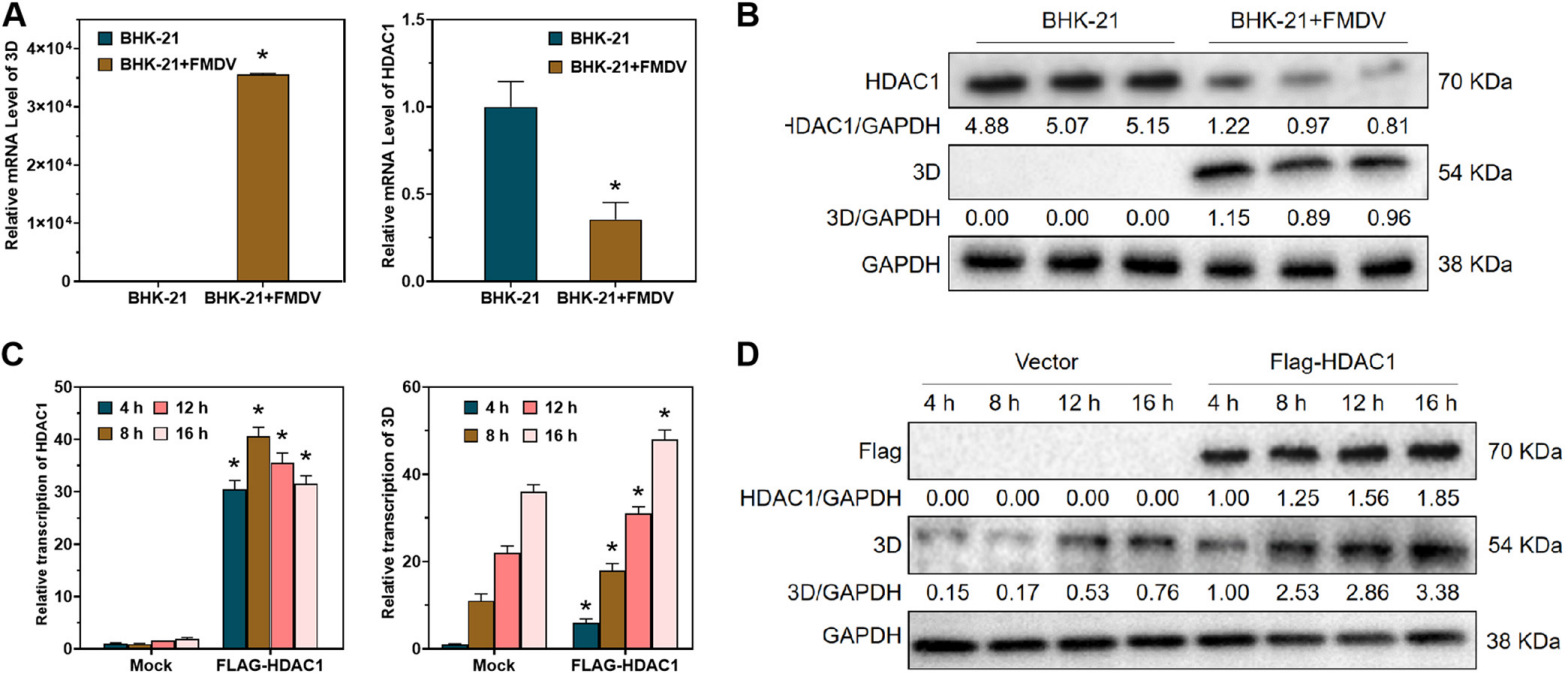
HDAC1 contributes to FMDV replication. (A) qRT-PCR was performed to detect the mRNA levels of viral 3D protein and host HDAC1 after FMDV infection. (B) Western blotting was performed to detect the protein levels of 3D and HDAC1 after FMDV infection. (C) qRT-PCR was performed to detect the mRNA levels of 3D and HDAC1 after plasmid-mediated overexpression of HDAC1. (D) Western blotting was performed to detect the protein levels of 3D and HDAC1 after overexpression of HDAC1. GAPDH was used as reference. *n* = 3. *, *P <* 0.05.

Our previous transcriptome sequencing (RNA-seq) results for BHK-21 cells with acute FMDV infection revealed that HDAC1 was differentially expressed after FMDV infection (32, 33). To examine the relationship between HDAC1 and FMDV infection further, we infected BHK-21 cells with FMDV at a titer of 10^3^ 50% tissue culture infective dose (TCID_50_) per milliliter and measured the expression of HDAC1 and viral protein. At 16 h postinfection, the cells were lysed, and RNA levels of the FMDV 3D protein and HDAC1 were detected using reverse transcription-quantitative PCR (qRT-PCR) (Fig. 1A). The results showed that the RNA levels of FMDV increased in the cells after infection, while the expression of HDAC1 was suppressed. The qRT-PCR results were consistent with Western blot results showing that 3D protein expression increased but HDAC1 was suppressed after viral infection (Fig. 1B). We overexpressed HDAC1 using an overexpression plasmid and examined the effect of HDAC1 overexpression on FMDV infection at 4 h, 8 h, 12 h, and 16 h postinfection. HDAC1 was significantly upregulated by the overexpression plasmid; however, the RNA level of FMDV 3D protein did not increase with increasing duration of infection in the HDAC1-overexpressing cells (Fig. 1C). Western blotting also showed that the protein level of 3D did not increase in the HDAC1-overexpressing cells from 4 h to 16 h postinfection (Fig. 1D). These results indicated that HDAC1 upregulated RNA levels and protein expression of FMDV at an early stage of infection, suggesting that HDAC1 plays a crucial role in FMDV infection and replication.

### The HDAC1-AKT-mTOR axis is inhibited by FMDV infection.

**FIG 2:**
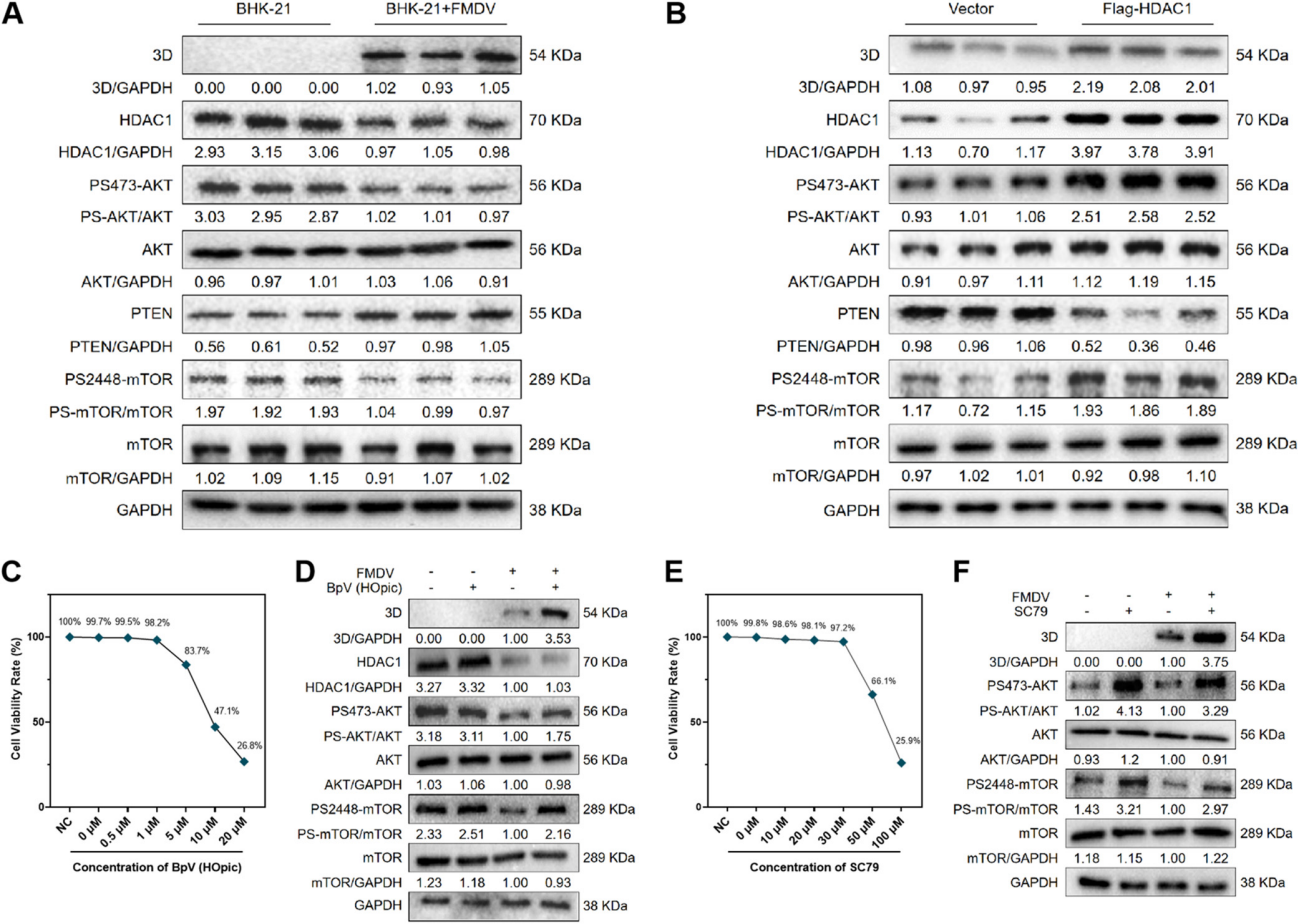
FMDV infection inhibited the HDAC1-AKT-mTOR axis. (A) Western blotting was performed to detect the protein levels of viral 3D protein, host HDAC1, and key proteins of the AKT-mTOR signaling pathway after FMDV infection. (B) Western blotting was performed to detect the protein levels of 3D, HDAC1, and key proteins of the AKT-mTOR signaling pathway after plasmid-mediated overexpression of HDAC1. (C) Cell counting kit-8 assay was performed to detect the cytotoxicity of different concentrations of bpV(HOpic) in BHK-21 cells. (D) Western blotting was performed to detect the protein levels of 3D, HDAC1, and key proteins of the AKT-mTOR signaling pathway after bpV(HOpic) treatment in BHK-21 cells. (E) Cell counting kit-8 assay was performed to detect the cytotoxicity of different concentrations of SC79 in BHK-21 cells. (F) Western blotting was performed to detect the protein levels of 3D, HDAC1, and key proteins of the AKT-mTOR signaling pathway after SC79 treatment in BHK-21 cells. GAPDH was used as a reference. *n* = 3.

The AKT-mTOR signaling pathway downstream of HDAC1 is critical during viral infection and may regulate the replication of a variety of RNA viruses. Therefore, to investigate the mechanism by which HDAC1 affects FMDV infection, we examined PTEN, AKT, and mTOR, the key proteins of the AKT-mTOR signaling pathway. Western blotting showed that FMDV infection inhibited the expression of HDAC1 and the phosphorylation of mTOR (Fig. 2A). When we upregulated HDAC1 in BHK-21 cells using an overexpression plasmid, we found that the viral 3D protein was activated and mTOR phosphorylation levels were increased (Fig. 2B). In addition, when we treated BHK-21 cells with 5 μM bpV(HOpic), a specific inhibitor of PTEN, or 30 μM SC79, an activator of AKT-mTOR signaling, we found that both compounds activated the AKT-mTOR signaling pathway. Cell counting kit-8 (CCK8) assays to assess the cytotoxicity of bpV(HOpic) and SC79 showed that both compounds exhibited low cytotoxicity (Fig. 2C and E). Both compounds also increased the phosphorylation of AKT and mTOR and the protein expression of FMDV 3D (Fig. 2D and F). These results reinforced that FMDV infection could influence the AKT-mTOR signaling pathway by affecting HDAC1 expression and that upregulation of the AKT-mTOR signaling pathway could positively regulate FMDV replication.

### CAD is induced by HDAC1 and regulates the replication of FMDV.

**FIG 3:**
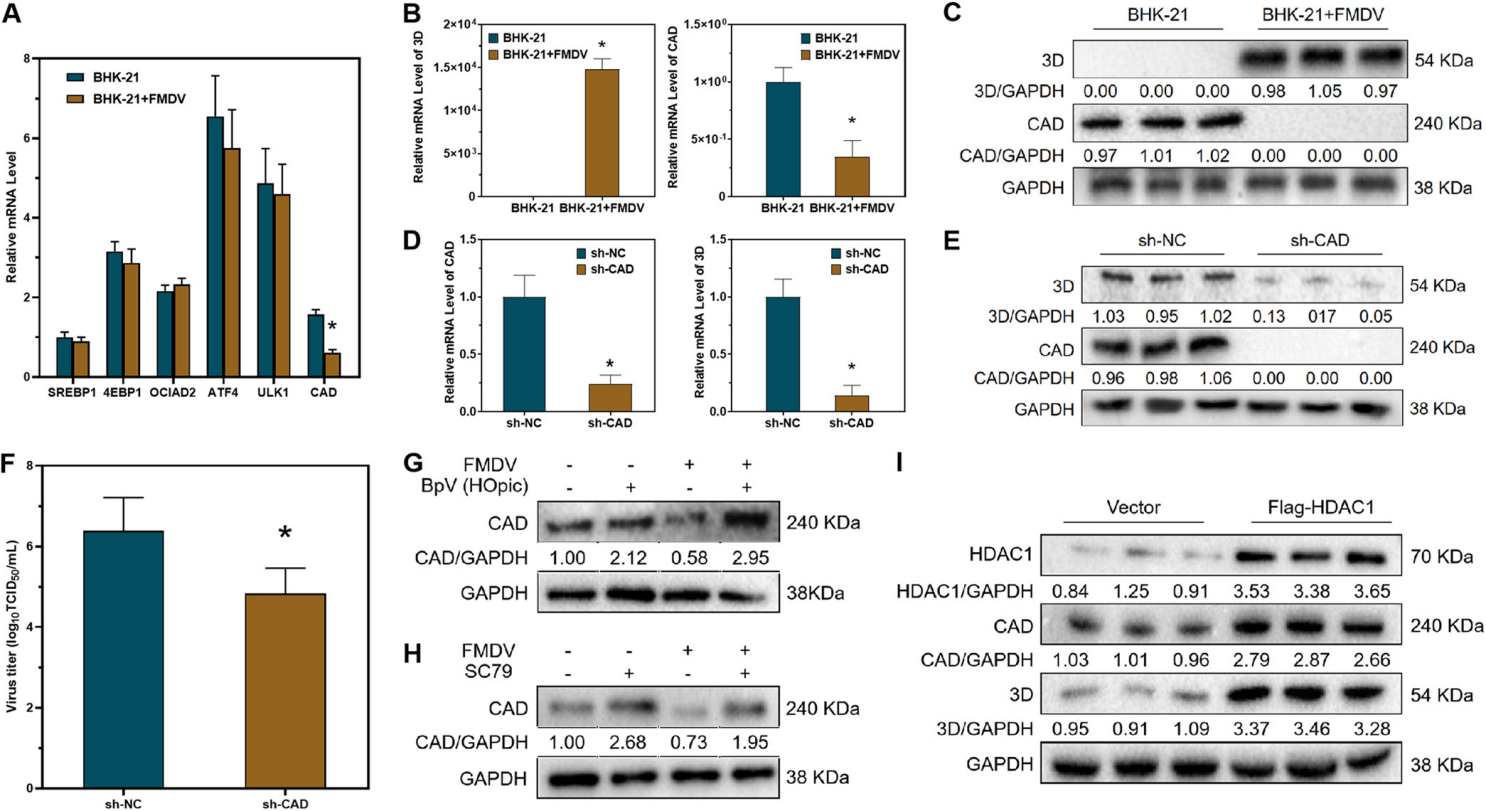
CAD was induced by HDAC1 and regulated the replication of FMDV. (A) qRT-PCR was performed to detect the mRNA levels of host proteins downstream of the AKT-mTOR signaling pathway after FMDV infection. (B) qRT-PCR was performed to detect the mRNA levels of viral 3D protein and host CAD after FMDV infection. (C) Western blotting was performed to detect the protein levels of 3D and CAD after FMDV infection. (D) qRT-PCR was performed to detect the mRNA levels of 3D and CAD after shRNA-induced downregulation of CAD. (E) Western blotting was performed to detect the protein levels of 3D and CAD after downregulation of CAD. (F) TCID_50_ assay was performed to detect the titer of infectious virus. (G) Western blotting was performed to detect the protein level of CAD after bpV(HOpic) treatment in BHK-21 cells. (H) Western blotting was performed to detect the protein level of CAD after SC79 treatment in BHK-21 cells. (I) Western blotting was performed to detect the protein levels of 3D, CAD, and HDAC1 after plasmid-mediated overexpression of HDAC1. GAPDH was used as a reference. *n* = 3. *, *P* < 0.05.

In the previous study of our laboratory, we performed RNA-seq to analyze genes that are differentially expressed after acute FMDV infection (30, 31). Some of the transcription factors downstream of the AKT-mTOR signaling pathway were differentially expressed (Fig. 3A), and the expression of CAD was suppressed after FMDV infection. We infected BHK-21 cells with FMDV and detected the expression of CAD in the cells, and the results were consistent with the RNA-seq results, showing significant downregulation of CAD RNA levels (Fig. 3B). Western blotting indicated that FMDV infection resulted in significant inhibition of CAD protein expression as well (Fig. 3C). In addition, we designed three pairs of CAD short hairpin RNAs (shRNAs) and selected the one with the best ability to disturb the expression of CAD (see Fig. S1 in the supplemental material). After shRNA knockdown of CAD in BHK-21 cells, we detected downregulation of FMDV 3D protein by qRT-PCR (Fig. 3D) and Western blotting (Fig. 3E). The titers of infectious virus in the supernatant were also decreased (Fig. 3F). Activation of the AKT-mTOR signaling pathway by bpV(HOpic) or SC79 resulted in upregulation of the CAD protein level (Fig. 3G and H). Finally, overexpression of HDAC1 in BHK-21 cells also resulted in upregulation of CAD (Fig. 3I). These results revealed that HDAC1 could upregulate CAD expression, and FMDV infection could suppress HDAC1 expression. The downregulation of HDAC1 leads to inhibition of the AKT-mTOR signaling pathway and, consequently, CAD expression.

### CAD interacts with the FMDV 2C protein in the viral replication complex.

**FIG 4:**
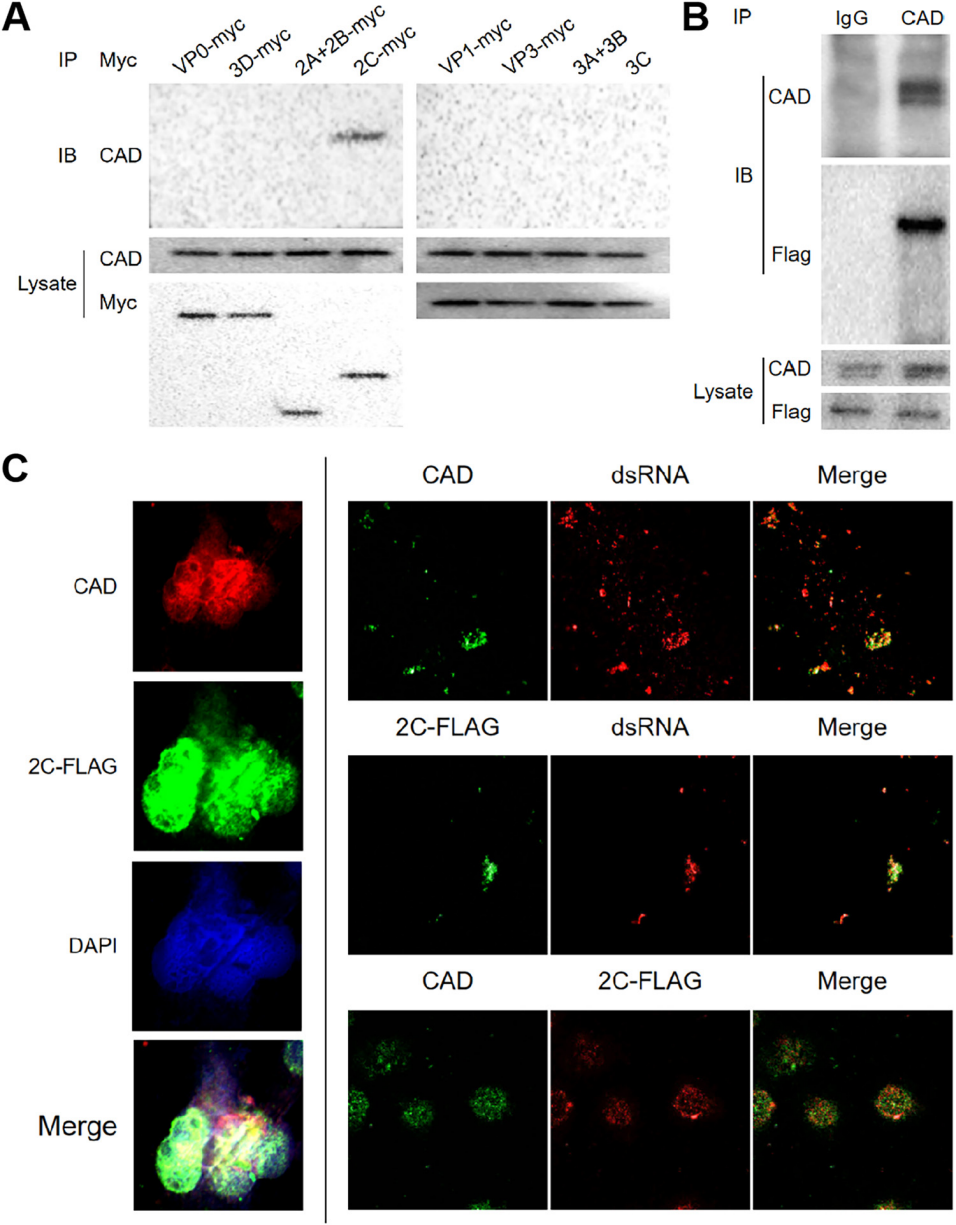
CAD interacts with FMDV 2C protein in the replication complex. (A) Coimmunoprecipitation and Western blotting were performed to detect the interaction of CAD with FMDV proteinVP_0_, 3D, 2A+2B, 2C, VP_1_, VP_3_, 3A+3B, and 3C. (B) Coimmunoprecipitation and Western blotting were performed to detect the interaction of CAD and FMDV 2C protein. (C) Immunofluorescence was performed to detect the colocalization of CAD, 2C, and FMDV double-stranded RNA. *n* = 3.

To investigate the mechanisms underlying the impact of CAD on FMDV replication, we first tested the interaction between CAD and different FMDV proteins using coimmunoprecipitation (co-IP) assays. We cotransfected cells with CAD and Flag-tagged viral proteins and performed co-IP using an antibody specific for CAD. The results showed that there did not appear to be protein-protein interactions between CAD and most viral proteins (Fig. 4A), but there was an interaction between CAD and the viral nonstructural protein 2C (Fig. 4A), so we performed a separate co-IP assay on CAD and 2C-Flag, and the results supported the conclusion that there was an interaction between the two proteins (Fig. 4B). The 2C protein is strongly associated with the replication complexes of some other picornaviruses. Therefore, we hypothesized that CAD regulates the function of the FMDV replication complex by interacting with FMDV 2C protein. Picornaviruses usually generate double-stranded replication intermediates during replication. We performed an immunofluorescence assay to examine the relationship among the CAD and 2C proteins and FMDV double-stranded RNA (dsRNA) (Fig. 4C). The fluorescence images of the uninfected cells are also presented in Fig. The results demonstrated that both CAD and 2C could colocalize with the double-stranded RNA in viral replication intermediates, suggesting that the two proteins could interact directly and are associated with the replication complex of the virus.

### The ACTase domain of CAD plays a critical role in FMDV replication.

**FIG 5:**
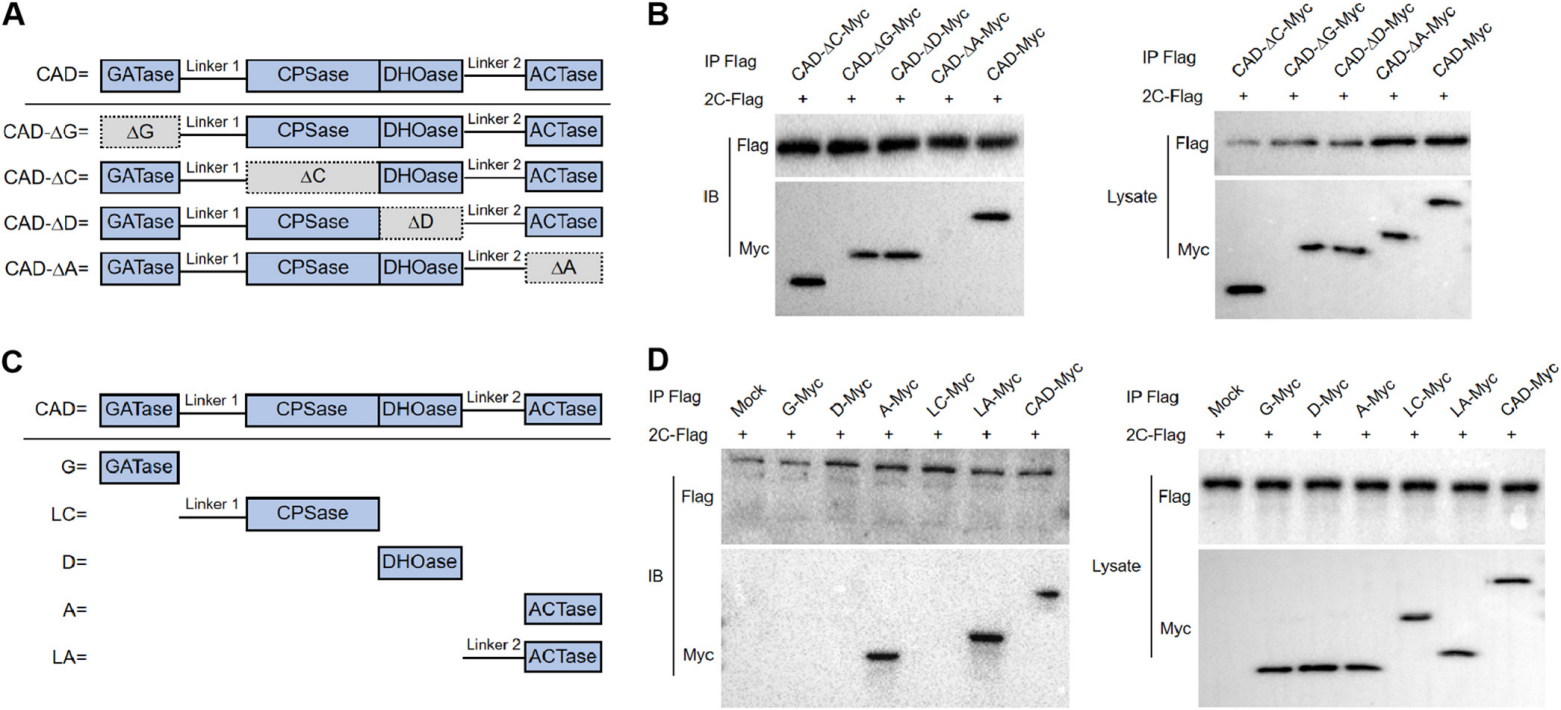
The ACTase domain of CAD plays a critical role in FMDV replication. (A) Schematic design of a truncated mutant with a single domain of CAD removed. (B) Coimmunoprecipitation and Western blotting were performed to detect the interaction of FMDV 2C protein and truncated CAD mutants with a single domain removed. (C) Schematic design of a truncated mutant containing only a single domain of CAD. (D) Coimmunoprecipitation and Western blotting were performed to detect the interaction of FMDV 2C protein and truncated CAD mutants containing only a single domain. *n* = 3.

To characterize the mechanism by which CAD interacts with the FMDV 2C protein, we constructed mutants with deletions of each of the four domains of CAD (Fig. 5A). We performed a co-IP assay using Flag-tagged antibody to detect the interaction between 2C and the mutants lacking each CAD domain. The results showed that mutants with deletion of the ACTase domain lost the interaction with the 2C protein (Fig. 5B). We also constructed CAD mutants containing only one of the four domains to further verify our results. Furthermore, to exclude the action of the linker that connects the CPSase and ACTase domains to the previous domains, we constructed mutants of the linker and its neighboring domains, named G-myc, D-myc, A-myc, LC-myc, and LA-myc (Fig. 5C). Flag-tagged antibody was used in co-IP assays, and the results showed that only the A-myc and LA-myc mutants could interact with FMDV 2C protein (Fig. 5D), suggesting that among the four domains of CAD, the ACTase domain is critical in the interaction with FMDV 2C.

### The regulation of viral replication by CAD is dependent on the pyrimidine synthesis function of CAD.

**FIG 6:**
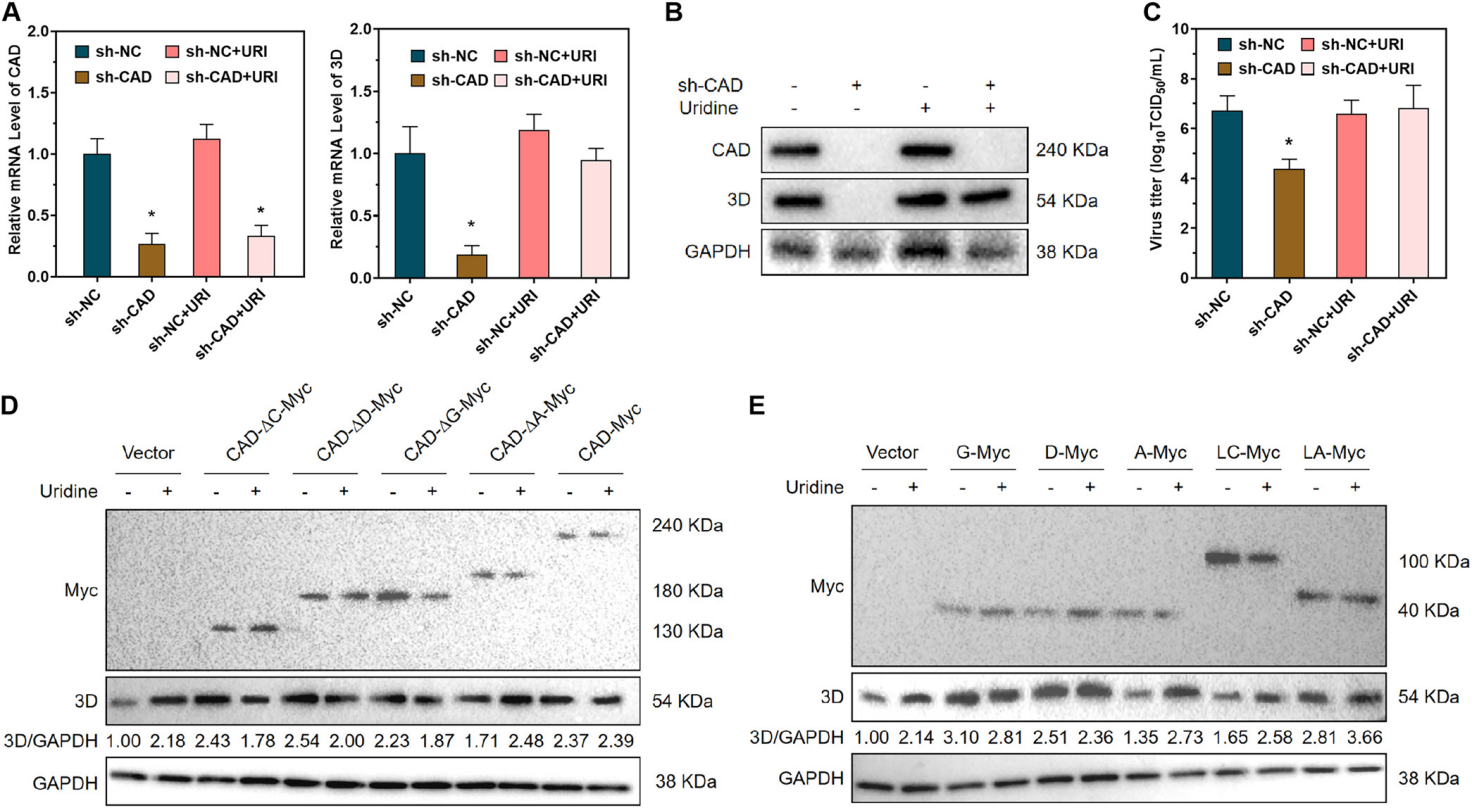
The regulation of viral replication by CAD is dependent on the pyrimidine synthesis function of CAD. (A) qRT-PCR was performed to detect the mRNA levels of CAD and viral 3D protein after shRNA-induced downregulation of CAD and uracil supplementation in BHK-21 cells. (B) Western blotting was performed to detect the protein levels of CAD and 3D after downregulation of CAD and uracil supplementation in BHK-21 cells. (C) TCID_50_ assay was performed to detect the titer of infectious virus. (D) Western blotting was performed to detect the protein level of 3D after plasmid-mediated overexpression of truncated CAD with a single domain removed and uracil supplementation in BHK-21 cells. (E) Western blotting was performed to detect the protein level of 3D after overexpression of a truncated CAD containing only a single domain and uracil supplementation in BHK-21 cells. GAPDH was used as a reference. *n* = 3. *, *P* < 0.05.

The ability of CAD to affect *de novo* pyrimidine synthesis and regulate RNA virus replication has been reported in many studies. Hence, we speculated that the inhibition of viral replication due to CAD knockdown could be reversed by supplementation with pyrimidines. When we supplemented cells with uracil after knocking down CAD, qRT-PCR and Western blot results showed that uracil supplementation reversed the inhibitory effect of CAD knockdown on the RNA and protein levels of 3D (Fig. 6A and B). The titers of infectious virus in the supernatant were also decreased (Fig. 6C). To examine whether the regulation of FMDV by the different domains of CAD is related to the function of CAD in *de novo* pyrimidine synthesis, we overexpressed each truncated CAD mutant in BHK-21 cells with or without uracil treatment and infected the cells with FMDV. Western blot analysis to detect viral protein levels showed that without the addition of uracil, CAD mutants with deletion of the GATase, CPSase, or DHOase domains could support the replication of FMDV better than CAD mutants with deletion of the ACTase domain (Fig. 6D). Similarly, we overexpressed mutants containing each domain alone, as well as mutants containing the linker and its adjacent domains, and treated the cells with uracil. The results showed that overexpression of the ACTase domain and the linker plus CPSase domains failed to increase the FMDV protein levels without uracil supplementation. In contrast, the viral protein levels in cells overexpressing the other domains did not depend on uracil supplementation (Fig. 6E). These results revealed that the inhibition of FMDV replication caused by knockdown of CAD could be reversed by uracil supplementation, indicating that the effect of CAD on FMDV replication is related to the function of CAD in pyrimidine synthesis.

## DISCUSSION

The small RNA viruses include many important human and animal pathogens. FMDV, one of the first small RNA viruses to be discovered, had a substantial negative impact on the breeding industry in the last century (1). The first model of persistent FMDV infection was established in 1985 (34) and laid the foundation for further investigation of the mutual adaptation of viruses and host cells. Recently, a high-resolution crystal structure of the FMDV 2C protein was resolved for the first time and was used to design an antiviral peptide that can dissociate 2C multimers, inhibit 2C ATPase activity, and disrupt 2C-induced lipid droplet aggregation (35). Further antigenic structural studies revealed a vulnerable region of lethal weakness in FMDV (36). In our laboratory, we shifted from early transcriptome studies with cell populations as samples to the single-cell level to study FMDV gene expression (30, 37, 38). In the first study to apply single-cell sequencing technology to cells with persistent FMDV infection, we showed that the MAPK-ERK signaling pathway and its downstream transcription factor, Fos, play an important role in persistent FMDV infection (33). On the other hand, analysis of transcriptome sequencing results revealed that knockdown of Cav1 promotes FMDV replication, whereas knockdown of Ccnd1 yields the opposite result (32). Easier and faster methods are needed to determine the expression and role of different genes during FMDV infection, and modern techniques for sequencing analysis make it possible to examine the role of cellular gene expression in FMDV-related studies.

The main function of histone deacetylases (HDACs) is to regulate cell proliferation, differentiation, metabolism, and immune responses by catalyzing the deacetylation of the amino-terminal lysine of histones, resulting in a densely coiled chromatin structure that inhibits gene transcription. Histone deacetylases 1 and 2 (HDAC1 and HDAC2, respectively) are the major histone deacetylases in eukaryotes and play an important role in the growth and metabolism of plants and animals. Histone deacetylation in plants promotes growth hormone, gibberellin, and jasmonic acid signaling by regulating key genes in several phytohormone pathways, and it also inhibits abscisic acid synthesis and signaling to promote fibroblast initiation (39). In cattle and mice, the histone deacetylase activity of HDAC1 and HDAC2 is required for zygotic genome activation (40). Although numerous studies have confirmed the important role of HDACs in the regulation of viral immunity *in vivo* and *in vitro*, few studies have looked at the role of HDACs in FMDV infection. Influenza virus NP proteins were shown to interact with HDAC1 *in vivo* and *in vitro*, and low expression of HDAC1 inhibited viral replication by reducing RNP polymerase activity and activating the TBK1-IRF3 signaling pathway (41). A recent study of the SARS-CoV-2 interactome suggests that that virus may interact with HDAC2, although the importance of the interaction is unclear. We found that overexpression of HDAC1 promotes FMDV replication at both the gene level and the protein level. This is consistent with previous results showing the effects of HDAC1 on other viral actions. It is noteworthy that homologous histone deacetylases may also play roles in antiviral immunity. For example, HDAC3 was shown to positively regulate type I interferon (IFN) by acting with FOXK1 and regulating STAT1 and STAT2 transcription, thus mediating antiviral innate immunity function of macrophages (42). The role of HDACs in different cells infected with various viruses needs to be explored in more detail.

The mammalian target of rapamycin (mTOR) kinase is an important molecule in signaling pathways involved in a variety of cellular activities. The PI3K-AKT-mTOR signaling pathway is one of the classical signaling pathways in which mTOR is involved (43). This pathway has important biological functions in cell growth, survival, proliferation, apoptosis, angiogenesis, and autophagy, and its disruption can cause a series of diseases (44–48). The important role of mTOR in innate immunity inextricably links the PI3K-AKT-mTOR pathway to viral infection. Hepatitis B virus has been shown to activate and exploit the mTOR signaling pathway to support its own replication in host cells (49). In a nephropathy model, mTOR inhibitors reduced the chance of cytomegalovirus infection in serologically positive kidney transplant recipients by improving T cell fitness (50). In another study, interference with mTOR activity using available drugs significantly reduced HIV replication in CD4 T cells from patients with undetectable viral loads (51). In recent years, the PI3K-AKT-mTOR pathway has also been shown to be relevant in coronavirus infection. Enhanced AKT phosphorylation upon SARS-CoV-2 infection induces alterations in RPS6KB1, a key regulator of mTOR-mediated translation, and the resulting RPS6KB1 activation reduces inhibition of mTOR, thereby promoting viral replication (52). Conversely, inhibition of the PI3K-AKT-mTOR pathway with the PI3K inhibitor pictilisib and the dual PI3K-mTOR inhibitor omipalisib inhibits SARS-CoV-2 replication (53). In exploring the mechanism by which HDAC1 affects FMDV replication, we looked at the downstream mTOR signaling pathway. We found that viral downregulation of HDAC1 triggered the downregulation of the AKT-mTOR pathway, which could be reversed by overexpression of HDAC1. Further experiments with inhibitors and activators confirmed that FMDV infection affects the AKT-mTOR pathway by regulating HDAC1 expression.

The mTORC1 protein complex, one of two major protein complexes involving mTOR, plays an essential role in the synthesis of proteins associated with cell proliferation and metabolism, and these activities are mediated by S6 kinases (S6Ks) and eukaryotic translation initiation factor 4E binding proteins (4E-Bps) (54). S6K1 phosphorylation activates CAD, which directs pyrimidine synthesis *ab initio*. To search for deeper mechanisms, we looked downstream of the AKT-mTOR pathway. Data from a previous transcriptome sequencing study showed that CAD was one of the downstream signals of the AKT-mTOR pathway that changed significantly after FMDV infection. Our results confirm that CAD plays an important role in FMDV genome replication and transcription; however, there are few studies of the potential role of CAD in viral invasion of host cells. CAD-related studies have mainly focused on the effects of activation and inhibition in cancer cells. It has been suggested that CAD activation mediated by S6K1 can be inhibited by inhibiting S6K1 activity, which also results in the inhibition of pseudorabies virus replication (55). We verified that viral effects on CAD are achieved through the AKT-mTOR pathway, HDAC1 can affect viral replication by regulating CAD, and there is a protein-protein interaction between the viral protein 2C and the host protein CAD. However, there is still much to explore regarding the specific mechanism by which CAD facilitates FMDV replication. It has been shown that CAD is recruited into Ebola virus inclusion bodies, ensuring that there are enough pyrimidines to replicate the genome of the virus (56). Replication transcription complexes (RTCs) are protein complexes that are synthesized by the cell's metabolic system and utilized by positive-stranded RNA viruses that do not have their own RNA replicase to complete viral RNA replication. For example, coronaviruses have been repeatedly shown to have an RTC structure, and a model has been proposed for their formation process (57). Therefore, it can be speculated that CAD acts as a link between upstream pathways, such as MAPK pathway and AKT-mTOR pathway, and formation of the FMDV RTC, and the next step can be to investigate the mechanism of interaction between CAD and the FMDV RTC. We used immunofluorescence to detect the localization of CAD, viral 2C protein, and viral double-stranded RNA in cells, and the results showed that there is a protein-protein interaction between viral 2C protein and CAD that most likely occurs in the FMDV replication complex. We also investigated the contribution of each structural domain of CAD to the interaction between CAD and FMDV using truncating CAD mutations. We found a strong interaction between the ACTase structural domain and 2C. The effect of CAD knockdown on viral replication could be reversed by complementation with pyrimidines. A clear grasp of the structure of the FMDV RTC might provide more ideas and possibilities regarding new targets for anti-FMDV drugs.

Several studies have shown that inhibition of pyrimidine biosynthesis can stimulate an innate antiviral response (58), but there was no detectable expression of IFN in our BHK-21 cells, suggesting that regulation of CAD expression influenced FMDV replication as a result of the function of CAD in pyrimidine metabolism. We found that the host proteins HDAC1 and CAD play a key role in the FMDV infection process. In mammalian cells, the pyrimidine biosynthetic pathway not only produces new nucleotides for nucleic acid synthesis, protein glycosylation, and cell membrane assembly but also supplies uridine nucleotides and derivatives that regulate key physiological processes such as lipid metabolism, central nervous system activity, and reproduction (59). It has been shown that regulatory elements of host lipid metabolism can influence viral replication by affecting the membrane assembly of the FMDV replication complex. It is worth investigating whether CAD can influence the FMDV life cycle by associating lipid metabolism with intermediates produced by the pyrimidine synthesis pathway. One study shows that CAD binding with VP1 virus may induce metabolic reprogramming of the host cell to promote viral replication (60). Furthermore, as a key regulator of glycosylation, the alterations in the pyrimidine synthesis pathway might affect the posttranslational modifications of host or viral proteins and thus viral replication. Highly glycosylated IFN-α has been shown to have potent antiviral and adjuvant effects against FMDV (61). Because BHK-21 cells lack IFN pathway activity, it remains to be investigated whether CAD can influence the IFN pathway through intermediate regulation of glycosylation via pyrimidine synthesis. With further investigation, the application of inhibitors of pyrimidine synthesis, such as leflunomide and teriflunomide, to antiviral research might lead to promising outcomes. The end product of CAD is UMP, which is interconvertible with uridine and can produce cytosine and thymine through a series of further biochemical reactions (62). Accordingly, we did not use cytidine, cytosine, purine, or other nucleosides as controls. Because even if these derivatives of the *de novo* pyrimidine synthesis regulated viral replication, it would still support our conclusion that CAD-dominated *de novo* pyrimidine synthesis is probably related to FMDV replication. Insufficiently, further experiments are still needed to determine the mechanism of these derivatives to regulate FMDV replication.

**FIG 7:**
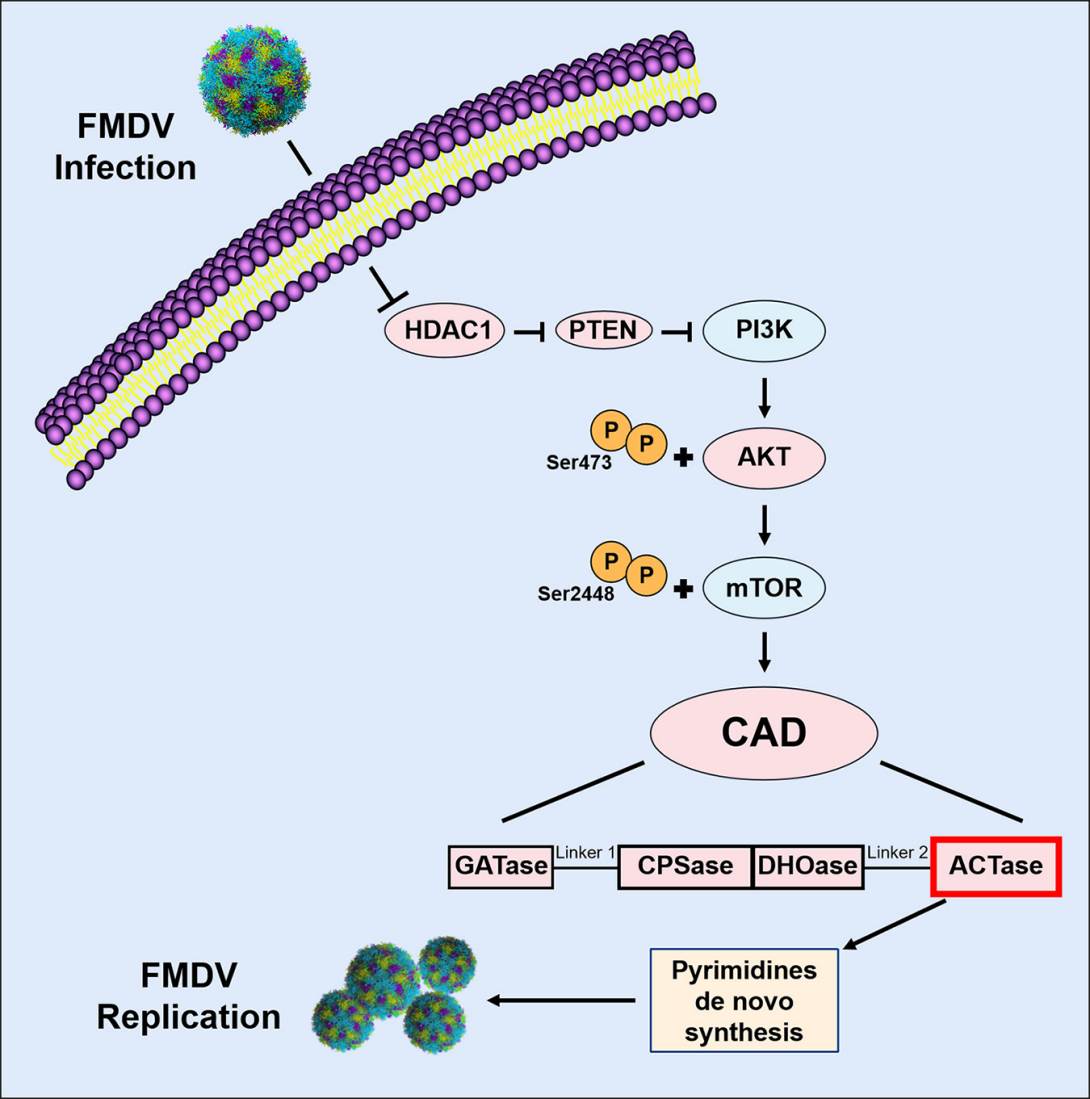
Schematic diagram depicting the regulation of FMDV replication by host CAD protein.

In conclusion, our results confirm that when FMDV infects cells, it inhibits the AKT-mTOR pathway by inhibiting HDAC1 and PTEN, which, in turn, prevents the downstream transcription factor CAD from interacting with FMDV, ultimately inhibiting FMDV replication (Fig. 7). Whether additional pathways are involved in this inhibition remains to be investigated. Our findings reveal a mechanism for the regulation of FMDV infection by the host protein CAD and show how host pyrimidine *de novo* synthesis is related to FMDV replication. We provide important theoretical implications for the future treatment and prevention of FMDV by regulating pyrimidine metabolism in host cells.

## MATERIALS AND METHODS

### Cells and viruses.

FMDV type O (Akesu/58/2002) was provided by the Lanzhou Veterinary Research Institute, Chinese Academy of Agricultural Sciences. Golden hamster kidney cells (BHK-21) were provided by CCTCC. BHK-21 cells were cultured in minimum essential medium (MEM) supplemented with 10% fetal bovine serum (Excell) and 1% streptomycin and penicillin. BHK-21 cells were cultured at 37°C with 5% CO_2_. The viral concentration of the FMDV stock was calculated by TCID_50_ assay.

### Cell transfection.

BHK-21 cells were cultured on a 12-well plate. After the density of cells reached 60%, solution A (1.2 μg pCMV-N-Flag plus 50 μL Opti-MEM medium per well) and solution B (1.2 μL Lipofectamine 2000 plus 50 μL Opti-MEM medium per well) were prepared separately. Five minutes after their preparation, solution A and solution B were mixed well. After 15 min, the culture medium in the wells of the 12-well plate was discarded, and the cells were washed with 1 mL sterile phosphate-buffered saline (PBS) three times. Then, 100 μL mixed solution A and B and 300 μL Opti-MEM were added to each well. The cells were then cultured at 37°C with 5% CO_2_ for 4 to 6 h. The solution was then discarded and replaced with MEM with 2% fetal bovine serum (FBS), and the cells were cultured at 37°C with 5% CO_2_ overnight.

### Virus infection and titer determination.

Frozen FMDV stock was melted in a 37°C water bath and mixed upside down. BHK-21 cells were grown to a density of 70%, at which point the MEM was discarded, and the cells were washed three times with PBS. FMDV was then added, and the cells were incubated at 37°C for 1 h. The FMDV solution was then discarded, and the cells were washed three times with PBS. Incubated at 37°C for 3 days, most of the cells showed cytopathic effects. The cells were then frozen at −80°C and melted at 37°C three times to make cell lysis complete. Viral titers were determined by TCID_50_ assay.

### RNA extraction and qRT-PCR.

Five hundred microliters of TRIzol was added to each well of a 12-well plate containing BHK-21 cell cultures and mixed well. The plate was then incubated at 4°C for 30 min. One hundred microliters of chloroform was then added and mixed well by vigorous shaking. The plate was then incubated at 4°C for 10 min. The cultures were then centrifuged at 12,000 × *g* for 15 min, and the supernatant was transferred to a new RNA-free centrifuge tube. An equal volume of isopropanol was added and mixed upside down, and the tube was incubated at 4°C for 10 min. The tube was then centrifuged at 12,000 × *g* for 10 min, and the supernatant was discarded. The precipitate was washed with 75% ethanol and centrifuged at 12,000 × *g* for 5 min. The supernatant was discarded, and the precipitate was dried thoroughly and then dissolved in 30 μL RNA-free water. cDNA was synthesized with a Hifair II 1st-strand cDNA synthesis kit (Yeasen). qRT-PCR was performed using Taq Pro universal SYBR qPCR master mix (Vazyme). Detailed instructions and procedures can be found in the corresponding protocols from the manufacturers. The primers for qRT-PCR are listed in Table 1. GAPDH (glyceraldehyde-3-phosphate dehydrogenase) was used as an internal reference gene.

**TABLE 1:** Primers used in this study^*a*^

Gene (reference)	Species	Sequence
*GAPDH* (32)	Golden hamster	F, AAGGCCATCACCATCTTCCA
		R, GCCAGTAGACTCCACAACATAC
*3D* (32)	Golden hamster	F, GAACACATTCTTTACACCAGGAT
		R, CATATCTTTGCCAATCAACATCAG
*CAD*	Golden hamster	F, CAGCTATGTACCTCCGCCC
		R, ACAACGACATCAGCGTAGCA

a The primers for CAD were designed by NCBI (https://www.ncbi.nlm.nih.gov/). The locations of the GAPDH primers are from 343 to 362 and from 408 to 429. The locations of the FMDV 3D primers are from 7173 to 7196 and from 7270 to 7293 in the genome of FMDV. The locations of the CAD primers are from 6050 to 6068 and from 6556 to 6575.

### Western blot analysis.

SDS-PAGE loading buffer and radioimmunoprecipitation assay (RIPA) lysis buffer (Beyotime) were mixed in advance. Cells were harvested with the mixed solution. Samples were resolved by 10% SDS-PAGE and transferred to a polyvinylidene difluoride (PVDF) membrane (Merck Millipore). The membrane was blocked with 5% milk (BD) in Tris-buffered saline with Tween 20 (TBST) for 1 h. The primary antibody was diluted with antibody diluent and used to detect the target protein on the membrane overnight at 4°C. The secondary antibody was then added and incubated for 2 h at room temperature. Antigens were detected with Immobilon Western chemiluminescent HRP substrate (Merck Millipore). The antibodies used in our experiment are listed in Table 2.

**TABLE 2:** Antibodies used in this study

Antibody	Company	Dilution ratio
GAPDH	Proteintech	1:2,000
FMDV 3D	Abiocenter	1:2,000
CAD	Affinity	1:2,000
DDDDK	Abclonal	1:200
Myc	Abclonal	1:2,000
PTEN	Huabio	1:1,000
p-AKT	ABclonal	1:500
AKT	ABclonal	1:500
mTOR	Cell Signaling Technology	1:500
p-mTOR	Cell Signaling Technology	1:500
HDAC1	Cell Signaling Technology	1:500
FMDV dsRNA	English and Scientific Consulting Kft	1:200
Goat anti-rabbit IgG	ABclonal	1:100
Goat anti-mouse IgG	ABclonal	1:100

### Coimmunoprecipitation.

BHK-21 cells in T-25 flasks were transfected at 80% cell density with 3 μg pCMV-2C-Flag for 24 h and then infected with FMDV. Sixteen hours after infection, the medium was discarded, and the cells were washed three times with PBS. Then, 1 mL Western and IP lysis solution (with protease inhibitor phenylmethylsulfonyl fluoride [PMSF] and cocktail) was added, and the cells were scraped off the culture flask and transferred into a 1.5-mL Eppendorf tube. The tube was incubated at 4°C on a rotary shaker for 20 min and then centrifuged for 10 min at 12,000 × *g* and 4°C. The sample was then diluted to 500 μL with Western and IP lysis solution. The antibody was diluted to 5 to 50 μg/mL using binding/washing buffer, and 400 μL diluted antibody was added to pretreated magnetic beads, mixed, and incubated on a flip mixer at 4°C for 2 h. The cell lysate was then added to the magnetic beads, mixed well by pipetting, and incubated on a rotary shaker for 30 min at room temperature. Magnetic separation was then performed on a magnetic stand, and the supernatant was transferred. The denaturing elution method was used to prepare the protein sample for subsequent Western blot detection.

### Immunofluorescence.

Glass crawlers were added to a 24-well plate with approximately 100,000 cells per well. After 24 h, the medium was discarded, and the cells were washed three times with Dulbecco’s PBS (DPBS). Then, 4% paraformaldehyde was added and fixed at room temperature for 30 min. The fixative was discarded, and the cells were washed three times with DPBS for 5 min. Then, 5% bovine serum albumin (BSA) in PBS was added and treated for 30 min. Primary antibody diluted with 1% BSA was then added and incubated at 4°C overnight. The mixture was then washed three times with PBS for 5 min, and secondary antibody diluted with 1% BSA was added and incubated at room temperature for 1 h. The secondary antibody was then discarded, and the mixture was washed three times with PBS for 5 min. DAPI (4′,6-diamidino-2-phenylindole) diluted with 1% BSA was used to stain the cells for 15 min, and the cells were washed again three times with PBS. The crawlers were then placed on a slide and observed with a confocal microscope (Zeiss).

### Statistical methods.

Data were expressed as the mean ± standard deviation. All statistical analyses were performed using SPSS version 16.0 (SPSS, Osaka, Japan). A *P* value of <0.05 was considered statistically significant.

## References

[1] Grubman MJ, Baxt B. 2004. Foot-and-mouth disease. Clin Microbiol Rev 17:465–493. doi:10.1128/CMR.17.2.465-493.2004. 15084510 PMC387408

[2] Fry EE, Stuart DI, Rowlands DJ. 2005. The structure of foot-and-mouth disease virus. Curr Top Microbiol Immunol 288:71–101. doi:10.1007/3-540-27109-0_4. 15648175

[3] Rodríguez Pulido M, Sáiz M. 2017. Molecular mechanisms of foot-and-mouth disease virus targeting the host antiviral response. Front Cell Infect Microbiol 7:252. doi:10.3389/fcimb.2017.00252. 28660175 PMC5468379

[4] Zhu Z, Li W, Zhang X, Wang C, Gao L, Yang F, Cao W, Li K, Tian H, Liu X, Zhang K, Zheng H. 2020. Foot-and-mouth disease virus capsid protein VP1 interacts with host ribosomal protein SA to maintain activation of the MAPK signal pathway and promote virus replication. J Virol 94:e01350-19. doi:10.1128/JVI.01350-19. PMC7000977 31694957

[5] Li D, Yang W, Yang F, Liu H, Zhu Z, Lian K, Lei C, Li S, Liu X, Zheng H, Shu H. 2016. The VP3 structural protein of foot-and-mouth disease virus inhibits the IFN-β signaling pathway. FASEB J 30:1757–1766. doi:10.1096/fj.15-281410. 26813975

[6] Wang D, Fang L, Li P, Sun L, Fan J, Zhang Q, Luo R, Liu X, Li K, Chen H, Chen Z, Xiao S. 2011. The leader proteinase of foot-and-mouth disease virus negatively regulates the type I interferon pathway by acting as a viral deubiquitinase. J Virol 85:3758–3766. doi:10.1128/JVI.02589-10. 21307201 PMC3126127

[7] Mason PW, Grubman MJ, Baxt B. 2003. Molecular basis of pathogenesis of FMDV. Virus Res 91:9–32. doi:10.1016/s0168-1702(02)00257-5. 12527435

[8] Belsham GJ. 2005. Translation and replication of FMDV RNA. Curr Top Microbiol Immunol 288:43–70. doi:10.1007/3-540-27109-0_3. 15648174

[9] García-Nuñez S, Gismondi MI, König G, Berinstein A, Taboga O, Rieder E, Martínez-Salas E, Carrillo E. 2014. Enhanced IRES activity by the 3′ UTR element determines the virulence of FMDV isolates. Virology 448:303–313. doi:10.1016/j.virol.2013.10.027. 24314661

[10] Wileman T. 2006. Aggresomes and autophagy generate sites for virus replication. Science 312:875–878. doi:10.1126/science.1126766. 16690857

[11] Li X, Wang M, Cheng A, Wen X, Ou X, Mao S, Gao Q, Sun D, Jia R, Yang Q, Wu Y, Zhu D, Zhao X, Chen S, Liu M, Zhang S, Liu Y, Yu Y, Zhang L, Tian B, Pan L, Chen X. 2020. Enterovirus replication organelles and inhibitors of their formation. Front Microbiol 11:1817. doi:10.3389/fmicb.2020.01817. 32973693 PMC7468505

[12] Sasaki J, Nagashima S, Taniguchi K. 2003. Aichi virus leader protein is involved in viral RNA replication and encapsidation. J Virol 77:10799–10807. doi:10.1128/jvi.77.20.10799-10807.2003. 14512530 PMC224959

[13] Moreno-Morcillo M, Grande-García A, Ruiz-Ramos A, del Cano-Ochoa F, Boskovic J, Ramón-Maiques S. 2017. Structural insight into the core of CAD, the multifunctional protein leading de novo pyrimidine biosynthesis. Structure 25:912–923.e5. doi:10.1016/j.str.2017.04.012. 28552578

[14] Sigoillot FD, Berkowski JA, Sigoillot SM, Kotsis DH, Guy HI. 2003. Cell cycle-dependent regulation of pyrimidine biosynthesis. J Biol Chem 278:3403–3409. doi:10.1074/jbc.M211078200. 12438317

[15] Hervé G. 2017. Structural insight into the core of CAD. Structure 25:819–820. doi:10.1016/j.str.2017.05.014. 28591622

[16] Lipscomb WN, Kantrowitz ER. 2012. Structure and mechanisms of Escherichia coli aspartate transcarbamoylase. Acc Chem Res 45:444–453. doi:10.1021/ar200166p. 22011033 PMC3276696

[17] Grande-García A, Lallous N, Díaz-Tejada C, Ramón-Maiques S. 2014. Structure, functional characterization, and evolution of the dihydroorotase domain of human CAD. Structure 22:185–198. doi:10.1016/j.str.2013.10.016. 24332717

[18] Löffler M, Carrey EA, Knecht W. 2020. The pathway to pyrimidines: the essential focus on dihydroorotate dehydrogenase, the mitochondrial enzyme coupled to the respiratory chain. Nucleosides Nucleotides Nucleic Acids 39:1281–1305. doi:10.1080/15257770.2020.1723625. 32043431

[19] Okesli A, Khosla C, Bassik MC. 2017. Human pyrimidine nucleotide biosynthesis as a target for antiviral chemotherapy. Curr Opin Biotechnol 48:127–134. doi:10.1016/j.copbio.2017.03.010. 28458037 PMC5659961

[20] Wittmann JG, Heinrich D, Gasow K, Frey A, Diederichsen U, Rudolph MG. 2008. Structures of the human orotidine-5′-monophosphate decarboxylase support a covalent mechanism and provide a framework for drug design. Structure 16:82–92. doi:10.1016/j.str.2007.10.020. 18184586

[21] Verrier ER, Weiss A, Bach C, Heydmann L, Turon-Lagot V, Kopp A, El Saghire H, Crouchet E, Pessaux P, Garcia T, Pale P, Zeisel MB, Sureau C, Schuster C, Brino L, Baumert TF. 2020. Combined small molecule and loss-of-function screen uncovers estrogen receptor alpha and CAD as host factors for HDV infection and antiviral targets. Gut 69:158–167. doi:10.1136/gutjnl-2018-317065. 30833451 PMC6943243

[22] Franks DM, Izumikawa T, Kitagawa H, Sugahara K, Okkema PG. 2006. C. elegans pharyngeal morphogenesis requires both de novo synthesis of pyrimidines and synthesis of heparan sulfate proteoglycans. Dev Biol 296:409–420. doi:10.1016/j.ydbio.2006.06.008. 16828468

[23] Arase T, Uchida H, Kajitani T, Ono M, Tamaki K, Oda H, Nishikawa S, Kagami M, Nagashima T, Masuda H, Asada H, Yoshimura Y, Maruyama T. 2009. The UDP-glucose receptor P2RY14 triggers innate mucosal immunity in the female reproductive tract by inducing IL-8. The. J Immunol 182:7074–7084. doi:10.4049/jimmunol.0900001. 19454705

[24] Sesma JI, Weitzer CD, Livraghi-Butrico A, Dang H, Donaldson S, Alexis NE, Jacobson KA, Harden TK, Lazarowski ER. 2016. UDP-glucose promotes neutrophil recruitment in the lung. Purinergic Signal 12:627–635. doi:10.1007/s11302-016-9524-5. 27421735 PMC5124001

[25] Dos Santos JC, Damen MS, Oosting M, de Jong DJ, Heinhuis B, Gomes RS, Araújo CS, Netea MG, Ribeiro-Dias F, Joosten LA. 2017. The NOD2 receptor is crucial for immune responses towards New World Leishmania species. Sci Rep 7:15219. doi:10.1038/s41598-017-15412-7. 29123157 PMC5680260

[26] de Bruyn M, Vermeire S. 2017. NOD2 and bacterial recognition as therapeutic targets for Crohn’s disease. Expert Opin Ther Targets 21:1123–1139. doi:10.1080/14728222.2017.1397627. 29096557

[27] Manning BD, Cantley LC. 2007. AKT/PKB signaling: navigating downstream. Cell 129:1261–1274. doi:10.1016/j.cell.2007.06.009. 17604717 PMC2756685

[28] Engelman JA. 2009. Targeting PI3K signalling in cancer: opportunities, challenges and limitations. Nat Rev Cancer 9:550–562. doi:10.1038/nrc2664. 19629070

[29] Fruman DA, Rommel C. 2014. PI3K and cancer: lessons, challenges and opportunities. Nat Rev Drug Discov 13:140–156. doi:10.1038/nrd4204. 24481312 PMC3994981

[30] Han L, Xin X, Wang H, Li J, Hao Y, Wang M, Zheng C, Shen C. 2018. Cellular response to persistent foot-and-mouth disease virus infection is linked to specific types of alterations in the host cell transcriptome. Sci Rep 8:5074. doi:10.1038/s41598-018-23478-0. 29568077 PMC5864922

[31] Li J, Han L, Hao Y, Yuan Y, Wang M, Xin X, Wang H, Yu F, Zheng C, Shen C. 2020. Comparative transcriptome analysis reveals different host cell responses to acute and persistent foot-and-mouth disease virus infection. Virol Sin 35:52–63. doi:10.1007/s12250-019-00155-8. 31512107 PMC7035396

[32] Han L, Yuan Y, Hu J, Li J, Zhu S, Yang P, Cheng A, Li X, Shen C. 2021. Next-generation sequencing sheds light on the interaction between virus and cell during foot-and-mouth disease virus persistent infection. Vet Microbiol 263:109247. doi:10.1016/j.vetmic.2021.109247. 34649012

[33] Yuan Y, Wang X, Li J, Han L, Du H, Sun Y, Yang P, Zhou Z, Gu M, Lu Y, Shen C. 2022. Single-cell sequencing yields insights in the evolution of foot-and-mouth disease virus persistent infection. Front Cell Infect Microbiol 12:940906. doi:10.3389/fcimb.2022.940906. 35873170 PMC9304859

[34] de la Torre JC, DáVila M, Sobrino F, Ortín J, Domingo E. 1985. Establishment of cell lines persistently infected with foot-and-mouth disease virus. Virology 145:24–35. doi:10.1016/0042-6822(85)90198-9. 2990100

[35] Zhang C, Yang F, Wojdyla JA, Qin B, Zhang W, Zheng M, Cao W, Wang M, Gao X, Zheng H, Cui S. 2022. An anti-picornaviral strategy based on the crystal structure of foot-and-mouth disease virus 2C protein. Cell Rep 40:111030. doi:10.1016/j.celrep.2022.111030. 35793627

[36] He Y, Li K, Wang L, Sun Z, Cao Y, Li P, Sun P, Bao H, Zhou S, Wang S, Bai X, Liu X, Zhao L, Fan X, Liu Z, Lu Z, Yang C, Lou Z. 2021. Structures of foot-and-mouth disease virus with bovine neutralizing antibodies reveal the determinant of intraserotype cross-neutralization. J Virol 95:e01308-21. doi:10.1128/JVI.01308-21. 34586859 PMC8610593

[37] Xin X, Wang H, Han L, Wang M, Fang H, Hao Y, Li J, Zhang H, Zheng C, Shen C. 2018. Single-cell analysis of the impact of host cell heterogeneity on infection with foot-and-mouth disease virus. J Virol 92:e00179-18. doi:10.1128/JVI.00179-18. 29444939 PMC5899210

[38] Fang H, Yuan B, Han L, Xin X, Wang H, Yu F, Zheng C, Shen C. 2017. Single-cell analysis reveals the relevance of foot-and-mouth disease virus persistence to emopamil-binding protein gene expression in host cells. Arch Virol 162:3791–3802. doi:10.1007/s00705-017-3546-3. 28916923

[39] Wei Z, Li Y, Ali F, Wang Y, Liu J, Yang Z, Wang Z, Xing Y, Li F. 2022. Transcriptomic analysis reveals the key role of histone deacetylation via mediating different phytohormone signalings in fiber initiation of cotton. Cell Biosci 12:107. doi:10.1186/s13578-022-00840-4. 35831870 PMC9277824

[40] Dang Y, Li S, Zhao P, Xiao L, Wang L, Shi Y, Luo L, Wang S, Wang H, Zhang K. 2022. The lysine deacetylase activity of histone deacetylases 1 and 2 is required to safeguard zygotic genome activation in mice and cattle. Development 149:dev200854. doi:10.1242/dev.200854. 35575026

[41] Chen L, Wang C, Luo J, Su W, Li M, Zhao N, Lyu W, Attaran H, He Y, Ding H, He H. 2017. Histone deacetylase 1 plays an acetylation-independent role in influenza A virus replication. Front Immunol 8:1757. doi:10.3389/fimmu.2017.01757. 29312300 PMC5733105

[42] Yang L, Chen S, Zhao Q, Pan C, Peng L, Han Y, Li L, Ruan J, Xia J, Yang H, Xu F, Cheng G. 2022. Histone deacetylase 3 contributes to the antiviral innate immunity of macrophages by interacting with FOXK1 to regulate STAT1/2 transcription. Cell Rep 38:110302. doi:10.1016/j.celrep.2022.110302. 35081346

[43] Laplante M, Sabatini DM. 2012. mTOR signaling in growth control and disease. Cell 149:274–293. doi:10.1016/j.cell.2012.03.017. 22500797 PMC3331679

[44] Saxton RA, Sabatini DM. 2017. mTOR signaling in growth, metabolism, and disease. Cell 168:960–976. doi:10.1016/j.cell.2017.02.004. 28283069 PMC5394987

[45] Watanabe R, Wei L, Huang J. 2011. mTOR signaling, function, novel inhibitors, and therapeutic targets. J Nucl Med 52:497–500. doi:10.2967/jnumed.111.089623. 21421716

[46] Forbes SA, Bindal N, Bamford S, Cole C, Kok CY, Beare D, Jia M, Shepherd R, Leung K, Menzies A, Teague JW, Campbell PJ, Stratton MR, Futreal PA. 2011. COSMIC: mining complete cancer genomes in the Catalogue of Somatic Mutations in Cancer. Nucleic Acids Res 39:D945–D950. doi:10.1093/nar/gkq929. 20952405 PMC3013785

[47] Ciuffreda L, Di Sanza C, Incani UC, Milella M. 2010. The mTOR pathway: a new target in cancer therapy. Curr Cancer Drug Targets 10:484–495. doi:10.2174/156800910791517172. 20384580

[48] Mayer IA, Arteaga CL. 2016. The PI3K/AKT pathway as a target for cancer treatment. Annu Rev Med 67:11–28. doi:10.1146/annurev-med-062913-051343. 26473415

[49] Wang X, Wei Z, Jiang Y, Meng Z, Lu M. 2021. mTOR signaling: the interface linking cellular metabolism and hepatitis B virus replication. Virol Sin 36:1303–1314. doi:10.1007/s12250-021-00450-3. 34580816 PMC8692646

[50] Kaminski H, Marseres G, Yared N, Nokin M-J, Pitard V, Zouine A, Garrigue I, Loizon S, Capone M, Gauthereau X, Mamani-Matsuda M, Coueron R, Durán RV, Pinson B, Pellegrin I, Thiébaut R, Couzi L, Merville P, Déchanet-Merville J. 2022. mTOR inhibitors prevent CMV infection through the restoration of functional αβ and γδ T cells in kidney transplantation. J Am Soc Nephrol 33:121–137. doi:10.1681/ASN.2020121753. 34725108 PMC8763189

[51] Planas D, Zhang Y, Monteiro P, Goulet J-P, Gosselin A, Grandvaux N, Hope TJ, Fassati A, Routy J-P, Ancuta P. 2017. HIV-1 selectively targets gut-homing CCR6^+^ CD4^+^ T cells via mTOR-dependent mechanisms. JCI Insight 2:e93230. doi:10.1172/jci.insight.93230. 28768913 PMC5543920

[52] Hekman RM, Hume AJ, Goel RK, Abo KM, Huang J, Blum BC, Werder RB, Suder EL, Paul I, Phanse S, Youssef A, Alysandratos KD, Padhorny D, Ojha S, Mora-Martin A, Kretov D, Ash PEA, Verma M, Zhao J, Patten JJ, Villacorta-Martin C, Bolzan D, Perea-Resa C, Bullitt E, Hinds A, Tilston-Lunel A, Varelas X, Farhangmehr S, Braunschweig U, Kwan JH, McComb M, Basu A, Saeed M, Perissi V, Burks EJ, Layne MD, Connor JH, Davey R, Cheng J-X, Wolozin BL, Blencowe BJ, Wuchty S, Lyons SM, Kozakov D, Cifuentes D, Blower M, Kotton DN, Wilson AA, Mühlberger E, Emili A. 2020. Actionable cytopathogenic host responses of human alveolar type 2 cells to SARS-CoV-2. Mol Cell 80:P1104–P1122.e9. doi:10.1016/j.molcel.2020.11.028. PMC7674017 33259812

[53] Klann K, Bojkova D, Tascher G, Ciesek S, Münch C, Cinatl J. 2020. Growth factor receptor signaling inhibition prevents SARS-CoV-2 replication. Mol Cell 80:164–174.Me4. doi:10.1016/j.molcel.2020.08.006. 32877642 PMC7418786

[54] Huang H, Long L, Zhou P, Chapman NM, Chi H. 2020. mTOR signaling at the crossroads of environmental signals and T-cell fate decisions. Immunol Rev 295:15–38. doi:10.1111/imr.12845. 32212344 PMC8101438

[55] Li Y, Yi L, Cheng S, Wang Y, Wang J, Sun J, Zhang Q, Xu X. 2021. Inhibition of canine distemper virus replication by blocking pyrimidine nucleotide synthesis with A77 1726, the active metabolite of the anti-inflammatory drug leflunomide. J Gen Virol 102:e001534. doi:10.1099/jgv.0.001534. 33416466

[56] Brandt J, Wendt L, Bodmer BS, Mettenleiter TC, Hoenen T. 2020. The cellular protein CAD is recruited into Ebola virus inclusion bodies by the nucleoprotein NP to facilitate genome replication and transcription. Cells 9:1126. doi:10.3390/cells9051126. 32370067 PMC7290923

[57] Yan L, Ge J, Zheng L, Zhang Y, Gao Y, Wang T, Huang Y, Yang Y, Gao S, Li M, Liu Z, Wang H, Li Y, Chen Y, Guddat LW, Wang Q, Rao Z, Lou Z. 2021. Cryo-EM structure of an extended SARS-CoV-2 replication and transcription complex reveals an intermediate state in cap synthesis. Cell 184:184–193.e10. doi:10.1016/j.cell.2020.11.016. 33232691 PMC7666536

[58] Lucas-Hourani M, Dauzonne D, Jorda P, Cousin G, Lupan A, Helynck O, Caignard G, Janvier G, André-Leroux G, Khiar S, Escriou N, Desprès P, Jacob Y, Munier-Lehmann H, Tangy F, Vidalain P-O. 2013. Inhibition of pyrimidine biosynthesis pathway suppresses viral growth through innate immunity. PLoS Pathog 9:e1003678. doi:10.1371/journal.ppat.1003678. 24098125 PMC3789760

[59] Connolly GP, Duley JA. 1999. Uridine and its nucleotides: biological actions, therapeutic potentials. Trends Pharmacol Sci 20:218–225. doi:10.1016/s0165-6147(99)01298-5. 10354618

[60] Cheng M-L, Chien K-Y, Lai C-H, Li G-J, Lin J-F, Ho H-Y. 2020. Metabolic reprogramming of host cells in response to enteroviral infection. Cells 9:473. doi:10.3390/cells9020473. 32085644 PMC7072837

[61] Kim A, Lee G, Hwang J-H, Park J-H, Lee MJ, Kim B, Kim S-M. 2022. BacMam expressing highly glycosylated porcine interferon alpha induces robust antiviral and adjuvant effects against foot-and-mouth disease virus in pigs. J Virol 96:e00528-22. doi:10.1128/jvi.00528-22. 35604219 PMC9215255

[62] Li G, Li D, Wang T, He S. 2021. Pyrimidine biosynthetic enzyme CAD: its function, regulation, and diagnostic potential. Int J Mol Sci 22:10253. doi:10.3390/ijms221910253. 34638594 PMC8508918

