# Supplementary material for: The Host Protein CAD Regulates the Replication of FMDV through the Function of Pyrimidines’ De Novo Synthesis
Source: J Virol. 2023 May 10;97(5):e00369-23. doi: 10.1128/jvi.00369-23 (PMC10231220; doi:10.1128/jvi.00369-23)
Supplement: Supplemental file 1 — Supplemental material. Download jvi.00369-23-s0001.pdf, PDF file, 0.3 MB [file jvi.00369-23-s0001.pdf]

The host protein CAD regulates the replication of FMDV through the function of pyrimidines de novo synthesis

Pu Yang[1,2†], Yuncong Yuan[1,2†], Yidan Sun[1,2], Bonan Lv[1,2], Hang Du[1,2], Zhou Zhou[1,2], Zhuang Yang[1,2], Xuemei Liu[1,2], Huimin Duan[1,2], Chao Shen[1,2*]

1 College of Life Sciences, Wuhan University, Wuhan 430072, China

2 China Center for Type Culture Collection, Wuhan University, Wuhan 430072, China

Corresponding author: Chao Shen (shenchao@whu.edu.cn)

† These authors contributed equally

Supplemental information

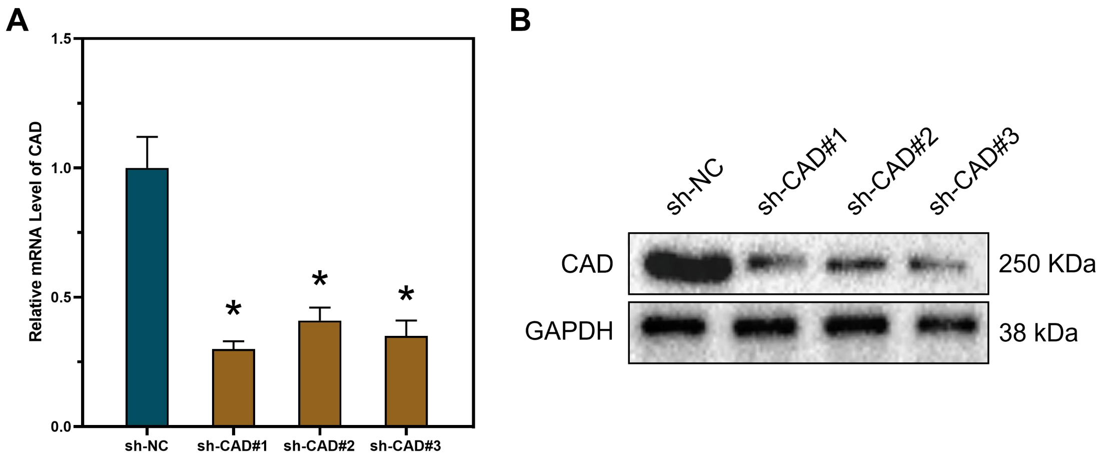

Figure S1. The knockdown of host protein CAD by shRNA.

(A) qRT-PCR was performed to detect the mRNA level of CAD after the down-regulation of CAD. (B) Western blot was performed to detect the protein level of CAD after the down-regulation of CAD. GAPDH was used as reference. n=3. *$P<0.05$.

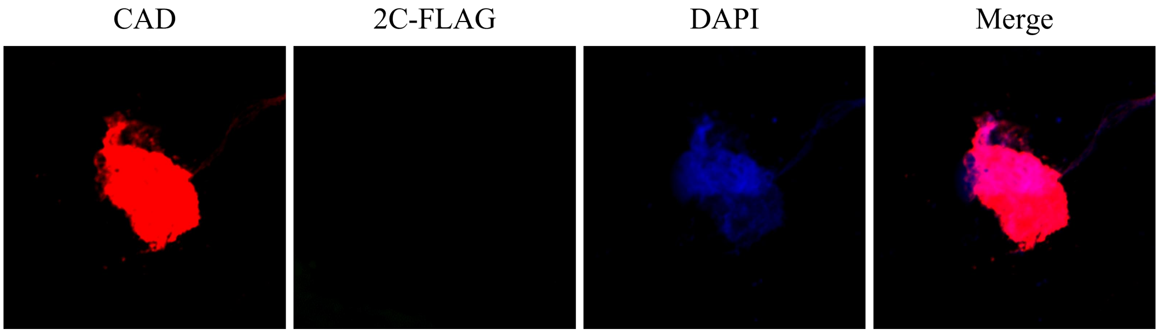

Figure S2. Immunofluorescence was performed to detect the co-location of CAD and FMDV 2C.
